# Role of Sirt3 in Differential Sex-Related Responses to a High-Fat Diet in Mice

**DOI:** 10.3390/antiox9020174

**Published:** 2020-02-20

**Authors:** Marija Pinterić, Iva I. Podgorski, Marijana Popović Hadžija, Ivana Tartaro Bujak, Ana Dekanić, Robert Bagarić, Vladimir Farkaš, Sandra Sobočanec, Tihomir Balog

**Affiliations:** 1 Division of Molecular Medicine, Ruđer Bošković Institute, 10000 Zagreb, Croatia; mpinter@irb.hr (M.P.); iskrinj@irb.hr (I.I.P.); mhadzija@irb.hr (M.P.H.); adekanic@irb.hr (A.D.); balog@irb.hr (T.B.); 2 Division of Experimental Physics, Ruđer Bošković Institute, 10000 Zagreb, Croatia; robert.bagaric@irb.hr (R.B.); vladimir.farkas@irb.hr (V.F.); 3 Division of Materials Chemistry, Ruđer Bošković Institute,10000 Zagreb, Croatia

**Keywords:** sirtuin 3, high fat diet, sex differences, mice, oxidative stress, metabolic stress

## Abstract

Metabolic homeostasis is differently regulated in males and females. Little is known about the mitochondrial Sirtuin 3 (Sirt3) protein in the context of sex-related differences in the development of metabolic dysregulation. To test our hypothesis that the role of Sirt3 in response to a high-fat diet (HFD) is sex-related, we measured metabolic, antioxidative, and mitochondrial parameters in the liver of Sirt3 wild-type (WT) and knockout (KO) mice of both sexes fed with a standard or HFD for ten weeks. We found that the combined effect of Sirt3 and an HFD was evident in more parameters in males (lipid content, glucose uptake, *pparγ*, *cyp2e1*, *cyp4a14*, Nrf2, MnSOD activity) than in females (protein damage and mitochondrial respiration), pointing towards a higher reliance of males on the effect of Sirt3 against HFD-induced metabolic dysregulation. The male-specific effects of an HFD also include reduced Sirt3 expression in WT and alleviated lipid accumulation and reduced glucose uptake in KO mice. In females, with a generally higher expression of genes involved in lipid homeostasis, either the HFD or Sirt3 depletion compromised mitochondrial respiration and increased protein oxidative damage. This work presents new insights into sex-related differences in the various physiological parameters with respect to nutritive excess and Sirt3.

## 1. Introduction

The metabolic syndrome is metabolic derangement involving a cluster of risk factors primarily associated with obesity, type two diabetes, and a high risk of cardiovascular events, but also with the development of inflammation, atherosclerosis, renal, liver and respiratory disease, cancer, and premature aging [1,2]. The prevalence of metabolic syndrome is dramatically on the rise in low- and mid-income countries and is one of the leading risks for global deaths representing serious threat to public health. The increase in caloric food intake or consumption of diets high in both fat and carbohydrates (also known as a “western diet”) along with physical inactivity leads to increased obesity, a key factor in the development of metabolic syndrome. Human metabolic syndrome can be effectively mimicked in rodent models using dietary intervention, such as high-fat diets (HFDs) [3]. The nutrient overload generated by an HFD in mice leads to a chronic increase in reactive oxygen species (ROS) production, which leads to oxidative stress (rev. in [1]). Oxidative stress is associated with the metabolic syndrome, but whether it is the cause or the consequence is a matter of debate. Nevertheless, an HFD is capable of functioning as a metabolic stressor causing mitochondrial dysfunction and other metabolic changes that contribute to pathological state and may mimic age-related pathology [4].

In most mammals, including humans, life expectancy differs between sexes. Females show lower incidence of some age-related pathologies linked with oxidative stress conditions and this sex-difference disappears after menopause, which led to the conclusion that this protection is attributed to the effect of sex hormones (rev in [5]). The important approach to study ageing and age-linked pathologies is to investigate sex dimorphism in defense to metabolic stressors. Historically, the impact of sex on metabolic status in mouse models has been neglected [6]. Indeed, most in vivo studies are focused on male mice. One of the reasons for a strong male sex bias is the belief that female mammals are intrinsically more variable than males due to the estrogen cycle. However, this has been disproven and without foundation [7]. While numerous papers examined the metabolic profiles of inbred mouse strain 129 [8,9,10], little attention has been paid to the impact of sex on the development of metabolic syndrome caused by HFD. 

Sirtuin 3 (Sirt3) is the only member of sirtuin family that has been linked to longevity in humans [11]. In addition, Sirt3 integrates cellular energy metabolism and various mitochondrial processes including ROS generation. The excessive production of ROS leads to oxidative stress, a crucial event that contributes to mitochondrial dysfunction and age-related pathologies [12]. Sirt3 plays a role in preventing metabolic syndrome [10], and is found to be upregulated in response to caloric restriction and exercise [13], while being downregulated with age, upon an HFD, and in diabetes [14]. Sirt3 is dispensable at a young age under homeostatic conditions but is essential under stress conditions or at an old age, making it a potential regulator of aging process. Although Sirt3 mediates oxidative stress suppression during caloric restriction [15], it remains to be elucidated whether Sirt3 may alleviate chronic oxidative stress initiated by excessive caloric intake in the form of an HFD. Many mitochondrial proteins are targets for deacetylation and activation by Sirt3, including proteins of oxidative phosphorylation, fatty acid oxidation, and the citric acid (TCA) cycle [16]. These data suggest that fatty acid metabolism and the TCA cycle are among the pathways that are tightly regulated by Sirt3. Moreover, Sirt3 is found to be regulated by a redox-sensitive, Keap1/Nrf2/ARE signaling axis, which facilitates transcription of Sirt3 and other antioxidant-response genes under stress conditions [17]. 

Little is currently known about Sirt3 expression in the context of sex-related differences in the development of metabolic syndrome. The understanding of sex differences in physiology and disease is of fundamental importance with regard to preventing metabolic disease. Therefore, our aim was to determine the role of Sirt3 in sex-related responses to a high-fat diet in 129S mice. In this study, we found that an HFD reduces hepatic Sirt3 expression only in wild-type (WT) males, while HFD-induced lipid accumulation is alleviated in Sirt3 knockout (KO) males, with impaired glucose uptake and increased reliance on fatty acids. Moreover, females had more efficient lipid metabolism but also displayed higher oxidative stress following an HFD in both genotypes with compromised mitochondrial respiration and increased protein oxidative damage. These data present new insights into sex-related differences in the metabolic parameters with respect to nutritive excess and Sirt3. 

## 2. Materials and Methods 

### 2.1. Animal Model and Experimental Design

Sirt3 WT (hereafter WT) and Sirt3 KO (hereafter KO) mice on a 129/SV background (stock no. 027975) were purchased from the Jackson Laboratory (Bar Harbor, ME, USA). WT and KO mice of both sexes at of 8 weeks of age, were fed with either a standard fat diet (SFD, 11.4% fat, 62.8% carbohydrates, 25.8% proteins; Mucedola, Settimo Milanese, Italy) or a high fat diet (HFD, 58% fat, 24% carbohydrates, 18% proteins; Mucedola, Settimo Milanese, Italy) during 10 weeks. The mice were age-matched and housed in standard conditions (3 mice per cage, 22 °C, 50–70% humidity, and a cycle of 12 h light and 12 h darkness). The animals were divided into 8 groups (6 mice per group): SFD-fed WT males, HFD-fed WT males, SFD-fed KO males, HFD-fed KO males, SFD-fed WT females, HFD-fed WT females, SFD-fed KO females, and HFD-fed KO females. Body weight was measured once a week, as well as glucose level, which was measured by glucometer (StatStrip Xpress-I, Nova Biomedical, GmbH, Mörfelden-Walldorf, Germany) in a blood drop taken from the tail vain. Before glucose measurements, mice were fasted for 6 h. After 10 weeks, mice were anesthetized by intraperitoneal injection of ketamine/xylazine (Ketamidor 10%, Richter pharma Ag, Wels, Austria; Xylazine 2%, Alfasan International, Woerden, Netherlands) and organs of interest were obtained and stored in liquid nitrogen until analysis. Animal experiments were done within the project funded by Croatian Science Foundation, project ID: IP-014-09-4533, approved on 01/09/2015. All procedures were approved by the Ministry of Agriculture of Croatia, (No: UP/I-322-01/15-01/25 525-10/0255-15-2 from 20 July 2015) and carried out in accordance with the associated guidelines EU Directive 2010/63/EU.

### 2.2. Histology and Oil Red O Staining 

A histological analysis of the liver sections from all experimental groups was performed in order to determine lipid accumulation caused by HFD. At the end of the feeding period, the anesthetized animals were perfused via the left ventricle of the heart with ice-cold phosphate-buffered saline (PBS; 137 mM NaCl, 2.7 mM KCl, 8 mM Na_2_HPO_4_, and 2 mM K_2_PO_4_, pH 7.4) for 2–3 min (to remove blood via the incised abdominal vena cava). Liver tissue was fixed by immersion in 4% paraformaldehyde in 0.1 M PBS (pH 7.4), left overnight at 4 °C, and was then washed with 1× PBS and cryoprotected in 30% sucrose in PBS until the sectioning on cryomicrotome at 8 μm. Fat vacuoles in hepatocytes of frozen sections were visualized by Oil Red O dye (Sigma Aldrich, St. Louis, MO, USA) prepared in propylene glycol (0.5% Oil Red O solution) according to the following protocol. The slides were dried for 60 min at room temperature (RT) and then fixed in ice-cold 10% formalin for 10 min followed by another 60 min of drying. The slides were then incubated for 5 min in absolute propylene glycol followed by staining in pre-warmed (60 °C) Oil Red O solution for 10 min and incubation for 5 min in 85% propylene glycol. Before staining with hematoxylin for 30 s, the slides were rinsed 2× in distilled water. After hematoxylin, the stained slides were thoroughly washed in running tap water followed by distilled water and mounted in mounting medium. An analysis of the stained liver sections was done using an Olympus BX51 microscope (Tokyo, Japan) with associated software analysis. The quantification of the lipid accumulation signal was done using ImageJ software (U.S. National Institutes of Health, Bethesda, MD, USA).

### 2.3. Total Lipid Extraction 

Total lipids were extracted from liver tissue according to a modified Folch procedure [18]. Briefly, 0.1 g of liver tissue was homogenized in MeOH, followed by the addition of 2 mL of CHCl_3_ and vigorous shaking. The mixture was filtered, and the remaining mixture was resuspended in CHCl_3_-MeOH (2:1). The mixture was filtered and washed again with fresh solvent, followed by washing the solution with 1.5 mL of 0.88% KCl and drawing off the aqueous layer using a Pasteur pipette. The washing step was repeated with 1.5 mL of KCl/MeOH (4:1, *v*/*v*), and the organic layer containing lipids was carefully transferred to a glass tube. A rotary evaporator (IKA Rotary evaporator RV 10 digital, Staufen, Germany) was used to remove the solvent, and the total lipids were determined by gravimetric analysis.

### 2.4. RNA Isolation and Quantitative Real-Time PCR Analysis

The TRIzol reagent (Invitrogen, Waltham, MA, USA) was used for total RNA extraction from a mouse liver (5% extract). The isolated RNA was treated with DNAse (TURBO DNA-free Kit, Thermo Fisher Scientific, Waltham, MA, USA) followed by reverse transcription using a High-Capacity cDNA Reverse Transcription Kit (Thermo Fisher Scientific) according to the manufacturer’s recommendations. For real-time PCR analysis, an ABI 7300 sequence detection system was used. To quantify the relative mRNA expression of *cyp2a4*, *cyp2e1*, *cyp4a14*, *hnf4α*, *pparα*, and *pparγ* in the livers of mice, the comparative C_T_ (^ΔΔ^C_T_) method according to the Taqman^®^ Gene Expression Assays Protocol (Applied Biosystems, Foster City, CA, USA) was used. The assays’ ID used for the analyses are shown in Supplementary Table S1. The data on the graphs are shown as the fold-change in gene expression, which is normalized to the endogenous reference gene (*β-actin*) and relative to the Sirt3 WT males fed with a SFD.

### 2.5. Protein Carbonylation

Protein carbonylation experiments were performed with an ELISA-based assay. Liver homogenates (5%) were prepared in PBS with protease inhibitors (Roche, Basel, Switzerland) and incubated for 1 h at RT with a lipid removal agent (13360-U, Sigma Aldrich, St. Louis, MO, USA), followed by centrifugation for 20 min at 16,000× *g*. A Pierce™ BCA Protein Assay Kit (Thermo Fischer Scientific) was used to determine the protein concentration, and 100 µL of 1 µg/µL lysate was incubated overnight at 4 °C using Maxisorb wells (Sigma Aldrich). 2,4-dinitrophenylhydrazine (DNPH; 12 µg/mL) was used for derivatization of adsorbed proteins, and rabbit anti-DNP primary (D9656, Sigma Aldrich) followed by goat anti-rabbit secondary antibody conjugated to HRP (NA934, GE Healthcare, Chicago, IL, USA) were used to detect the derivatized dinitrophenol (DNP)-carbonyl. Enzyme substrate 3,3′,5,5′-tetramethylbenzidine (Sigma Aldrich) was added into samples and incubated until color developed, followed by stopping the reaction using 0.3 M H_2_SO_4_. The absorbance was measured at 450 nm on a microplate reader (Bio-Tek Instruments, Winooski, VT, USA).

### 2.6. Analysis of Antioxidative Enzyme Activities

Antioxidative enzyme activities were analyzed in liver tissue lysates homogenized in PBS supplemented with protease inhibitors (Roche, Basel, Switzerland) using an ice-jacketed Potter-Elvehjem homogenizer (Thomas Scientific, Swedesboro, NJ, USA), at 1300 rpm. Superoxide dismutase (SOD) activities were determined measuring inhibition of the xanthine/xanthine oxidase-mediated reduction of 2-(4-iodophenyl)-3-(4-nitrophenyl)-5-phenyltetrazolium chloride (I.N.T.) using a Ransod kit (Randox Laboratories, Crumlin, UK) according to the manufacturer’s recommendations. This reaction is inhibited by the conversion of the superoxide radical to hydrogen peroxide and oxygen as a consequence of SOD activity. A total of 4 mM of KCN for 30 min was used to selectively inhibit CuZnSOD activity and obtain the MnSOD activity. CuZnSOD activity was obtained by subtracting the MnSOD activity from the total SOD activity. The Catalase (Cat) activity was done, as previously described [19], by measuring the change in absorbance (at 240 nm) in the reaction mixture during the interval of 30 s following sample addition. The final concentrations of 10 mM H_2_O_2_ and 50 mM PBS (pH 7.0) were used. Glutathione peroxidase (Gpx) activity was measured at 340 nm, as previously described [20], using a RANSEL kit (Randox Laboratories). In kit, a decrease in absorbance at 340 nm is accompanied by NADPH oxidation to NADP^+^ as an indirect measure of Gpx activity.

### 2.7. Protein Isolation and Western Blot Analysis

Liver samples were homogenized in an ice-cold lysis buffer (RIPA buffer supplemented with protease inhibitors (Roche)) using an ice-jacketed Potter-Elvehjem homogenizer (1300 rpm). Liver homogenates (5%) were centrifuged at 2000× *g* for 15 min at 4 °C, and the supernatant was sonicated (3 × 30 s) and centrifuged at 16,000× *g* for 20 min at 4 °C. The resulting lysate was transferred to a new tube and the protein concentration was estimated by the Pierce™ BCA Protein Assay Kit (Thermo Fischer Scientific). Proteins (40 µg/µL) were resolved by SDS-PAGE and were electrotransferred onto a PVDF membrane (Roche, Basel, Switzerland). Membranes were blocked in a I-Block™ Protein-Based Blocking Reagent (Invitrogen, Waltham, MA, USA) for 1 h at RT and were incubated with primary polyclonal or monoclonal antibodies overnight at 4 °C. For chemiluminiscence detection, an appropriate horseradish peroxidase (HRP)-conjugated secondary antibody was used. The list of primary and secondary antibodies is in Supplementary Table S2. AmidoBlack (Sigma Aldrich) was used for total protein normalization. The Alliance 4.7 Imaging System (UVITEC, Cambridge, UK) was used for the detection of immunoblots using an enhanced chemiluminscence kit Kit (Thermo Fischer Scientific).

### 2.8. Mitochondria Isolation and Oxygen Consumption

Mice liver mitochondria were isolated by differential centrifugation as described previously [21], with the following modification: liver was homogenized at a ratio of 100 mg tissue/mL of isolation buffer (10% liver homogenate). Isolated mitochondria were kept in the isolation buffer (250 mM sucrose, 2 mM EGTA, 0.5% fatty acid-free BSA, 20 mM Tris-HCl, pH 7.4) until the experiment on the Clark-type electrode (Oxygraph, Hansatech Instruments Ltd., Pentney, UK) in an airtight 1.5 mL chamber at 35°C. The protein concentration was determined with a Pierce™ BCA Protein Assay Kit. For the determination of oxygen consumption, mitochondria (800 µg protein) were resuspended in a 500 μL respiration buffer (200 mM sucrose, 20 mM TrisHCl, 50 mM KCl, 1 mM MgCl_2_·6H_2_O, 5 mM KH_2_PO_4_, pH 7,0). Complex I assessment samples were incubated with 2.5 mM glutamate and 1.25 mM malate. Mitochondrial respiration was accelerated by the addition of 2 mM ADP for state 3 respiration measurements. Then, ATP synthesis was terminated by adding 5 μg/mL of oligomycin to achieve state 4 rate. To inhibit the mitochondrial respiration, 2 μM antimycin A was used. Oxygen uptake is calculated in nmol/min/mg protein.

### 2.9. PET Analysis

For ^18^FDG-microPET imaging, animals have been anesthetized in induction chamber with 4% isoflurane (Forane, Abbott Laboratories, Chicago, IL, USA) and intraperitoneally injected with 100–200 µL of solution containing 25 MBq of radiotracer [^18^F] fluoro-2-deoxy-2-d-glucose (^18^FDG). To avoid the influence of warming on ^18^FDG biodistribution in mice injected intravenously, in our experiments we used the model of intraperitoneal FDG administration described in [22]. ^18^FDG-microPET imaging, along with ^18^FDG liver uptake data analysis, was performed according to our previous model [23]. The co-registration of PET images was made in PMOD FUSION software mode (PMOD Technologies LLC, Zürich, Switzerland). The final result is given in standardized uptake value units (SUV).

### 2.10. Statistical Analysis

For the statistical analysis of data, SPSS for Windows (17.0, IBM, Armonk, NY, USA) was used. A Shapiro–Wilk test was used before all analyses to test the samples for normality of distribution. Since all data followed normal distribution, parametric tests for multiple comparisons were performed: a student’s *t*-test for comparisons between males and females, and a two-way ANOVA for the interaction effect of Sirt3 × diet within each sex. If a significant interaction was observed, all pairwise comparisons were made between groups, using Tukey’s post-hoc test with Bonferroni’s correction. Significance was set at *p* < 0.05. On graphical displays, the indicator of the differences between males and females was marked as x; the indicator of differences between SFD and HFD (the effect of diet) was marked as a letter (a, b, etc.); the indicator of differences between WT and KO (the effect of Sirt3) was marked as *.

## 3. Results

### 3.1. HFD Reduces Hepatic Sirt3 Protein Expression in Males Only

**Figure 1:**
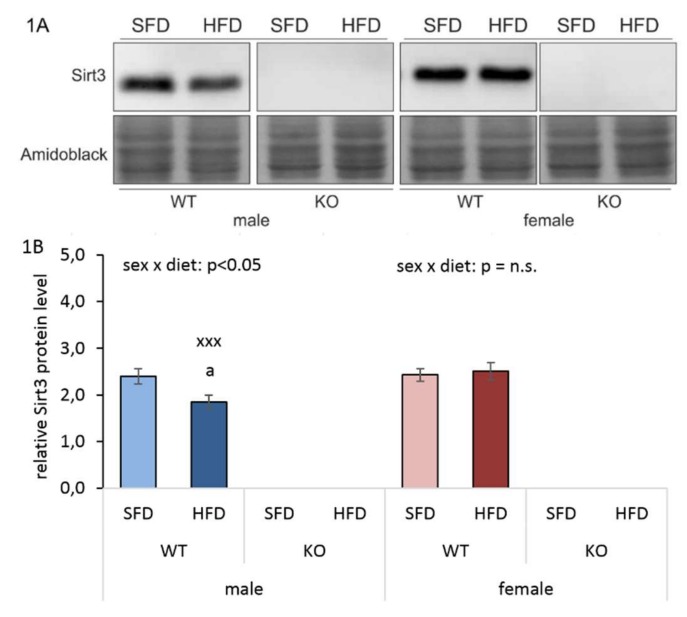
Hepatic Sirtuin 3 (Sirt3) protein expression in Sirt3 wild-type (WT) and knockout (KO) mice fed with a standard fat diet (SFD) or a high fat diet (HFD) for 10 weeks. (**A**) A representative immunoblot of the hepatic Sirt3 protein expression level. (**B**) A graphical display of the averaged densitometry values of immunoblots in (A). Males: HFD-fed vs. SFD-fed WT mice (^a^ *p* < 0.001); Females: no changes. Males vs. females: HFD-fed WT mice (^xxx^ *p* < 0.001). The data are shown as mean ± SD. *n* = 6. Amidoblack was used as a loading control.

To investigate if the hepatic expression of Sirt3 was altered in a sex- or diet-dependent manner, we first detected Sirt3 protein expression in all groups. Expectedly, in KO mice, Sirt3 protein level was undetectable. In WT mice, HFD partially (24%) reduced Sirt3 protein expression in males but did not affect Sirt3 in females. Therefore, HFD-fed males had lower Sirt3 expression than females (Figure 1A,B). These data suggest that ten weeks of HFD feeding reduces Sirt3 protein expression in a male-specific manner.

### 3.2. HFD Has no Infuence on Body Weight and Glucose Level

**Figure 2:**
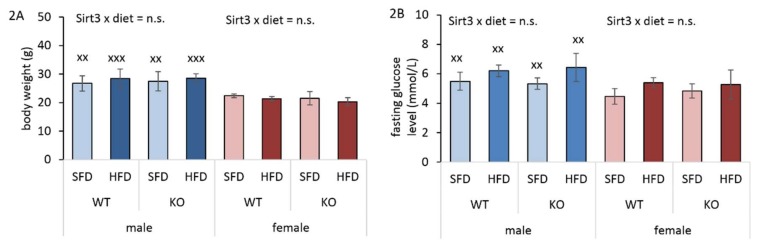
Body weight and fasting glucose level in Sirt3 wild-type (WT) and knockout (KO) mice fed with a standard fat diet (SFD) or a high fat diet (HFD) for 10 weeks. (**A**) A graphical display of body weight. Males: no changes. Females: no changes. Males vs. females: SFD-fed mice (^xx^ *p* < 0.01); HFD-fed mice (^xxx^ *p* < 0.001). (**B**) A graphical display of fasting glucose levels. Males: no changes. Females: no changes. Males vs. females: ^xx^ *p* < 0.01. The data are shown as mean ± SD. *n* = 6 per group.

It has been shown that the decreased level of Sirt3 contributes to impaired glucose metabolism [24], and since we noticed reduced Sirt3 expression upon HFD in WT males, we next tested whether differential Sirt3 expression and HFD would affect body weight and fasting glucose levels. After 10 weeks of feeding, treatment with an HFD did not affect whole body weight or glucose level in either sex, irrespective of Sirt3 (Figure 2A,B, Figure S1A,B). However, at the end of the 10th week, males had a higher body weight and glucose level compared to female mice (Figure 2A,B).

### 3.3. Differential Hepatic Fat Accumulation in Males Upon HFD Depends on Sirt3

**Figure 3:**
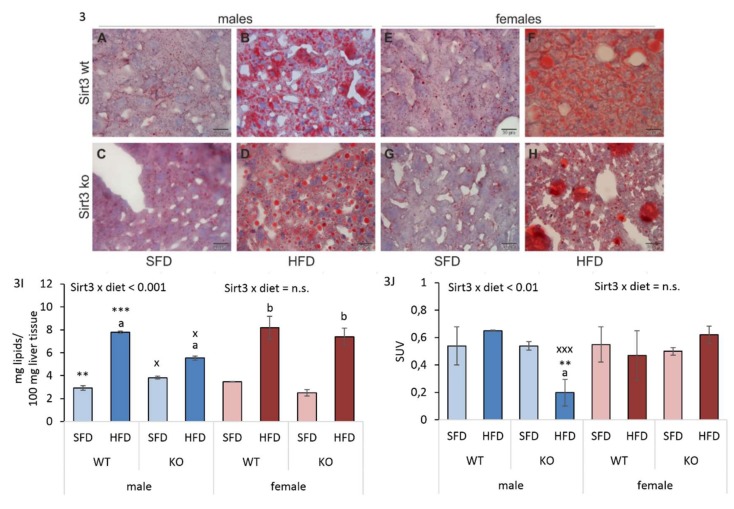
Sex-related differences in hepatic lipid accumulation and glucose (^18^FDG) uptake in Sirt3 wild-type (WT) and knockout (KO) mice of both sexes fed with a standard fat diet (SFD) or a high fat diet (HFD) for 10 weeks. (**A**–**H**) Liver cryo-sections stained with Oil Red O. Representative images show lower fat content in mice fed with a SFD (A, C, E, G), and promoted lipid accumulation in HFD-fed mice of both sexes and genotypes (B,D,F,H). (**I**) A graphical display of total lipid content in liver samples. Males: HFD-fed vs. SFD-fed mice (^a^ *p* < 0.001); SFD-fed WT vs. KO mice (** *p* < 0.01); HFD-fed WT vs. KO mice (*** *p* < 0.001). Females: HFD-fed vs. SFD-fed mice (^b^ *p* < 0.001). Males vs. females: SFD-fed KO mice (^x^ *p* < 0.05); HFD-fed KO mice (^x^ *p* < 0.05). (**J**) A graphical display of hepatic glucose uptake expressed as standardized uptake value (SUV). Males: SFD-fed vs. HFD-fed KO mice (^a^ *p* < 0.001); HFD-fed WT vs. KO mice (** *p* < 0.01). Females: no changes. Males vs. females: HFD-fed KO mice (^xxx^ *p* < 0.001). Data are shown as mean ± SD. *n* = 3–4 per group. scale bar = 20 μm.

While glucose levels showed a male-specific pattern, the interesting fact was that the HFD failed to cause weight gain compared to SFD in both sexes. To investigate whether HFD-fed mice developed a fatty liver, we determined the hepatic accumulation of lipids. Expectedly, fat vacuoles were predominately present in the livers of HFD-fed mice of both genotypes, confirming that HFD treatment caused lipid accumulation in mice livers (Figure 3A–H, Supplementary Figure S2). Folch extraction was performed to measure lipid content in larger parts of liver tissue. In males, an HFD caused significant lipid accumulation in both WT and KO mice. In SFD conditions, WT males had less, and, in HFD conditions, WT males had more hepatic lipids than KO males. In females, an HFD also induced significant lipid accumulation regardless of Sirt3. While in WT mice there were no differences in lipid content between males and females, more lipids were observed in SFD-fed, and less in HFD-fed KO males compared to KO females (Figure 3I). These results suggest that, in males, the hepatic lipid accumulation was partially influenced by Sirt3.

### 3.4. HFD Reduces Hepatic Glucose Uptake in Sirt3 KO Males

Because the presence of non-alcoholic fatty liver disease (NAFLD) is closely associated with decreased glucose metabolism, we determined hepatic glucose uptake using ^18^F-FDG PET. In KO males, an HFD significantly reduced glucose uptake. Also, HFD-fed KO males had lower glucose uptake than WT mice. On the contrary, in females, an HFD had no effect on glucose uptake across groups. Reduced hepatic glucose uptake in HFD-fed KO males resulted in significantly lower values compared to HFD-fed KO females. These data indicate the importance of Sirt3 in the maintenance of hepatic glucose uptake following HFD, but only in males (Figure 3J).

### 3.5. HFD Further Suppresses the Male-Specific Reduction of Hepatic hnf4α, pparα, pparγ, and cyp2a4 mRNA Level

Since it was observed that an HFD causes lipid accumulation in liver, we wanted to investigate several important genes involved in the lipid metabolism that are known to be expressed in sex-related manner and may be responsible for the observed phenotype, such as *hnf4α, pparα, pparγ, cyp2a4, cyp2e1,* and *cyp4a14*.

**Figure 4:**
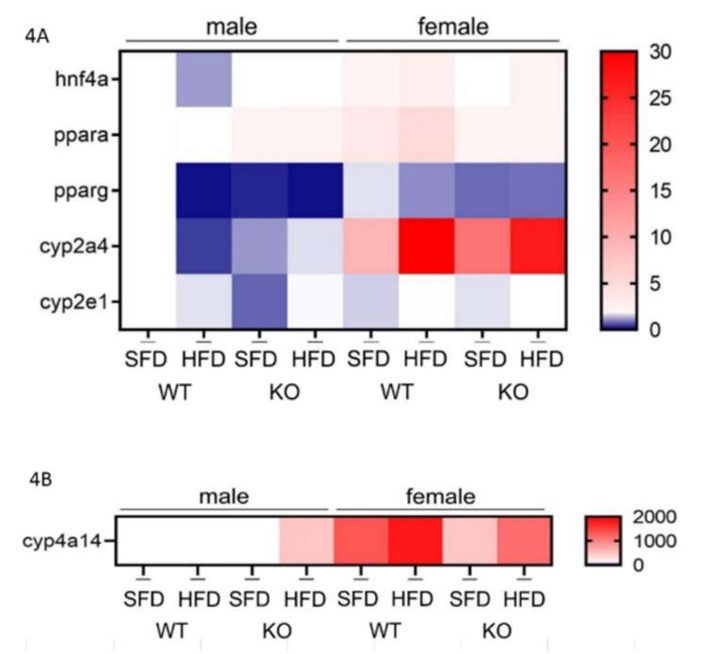

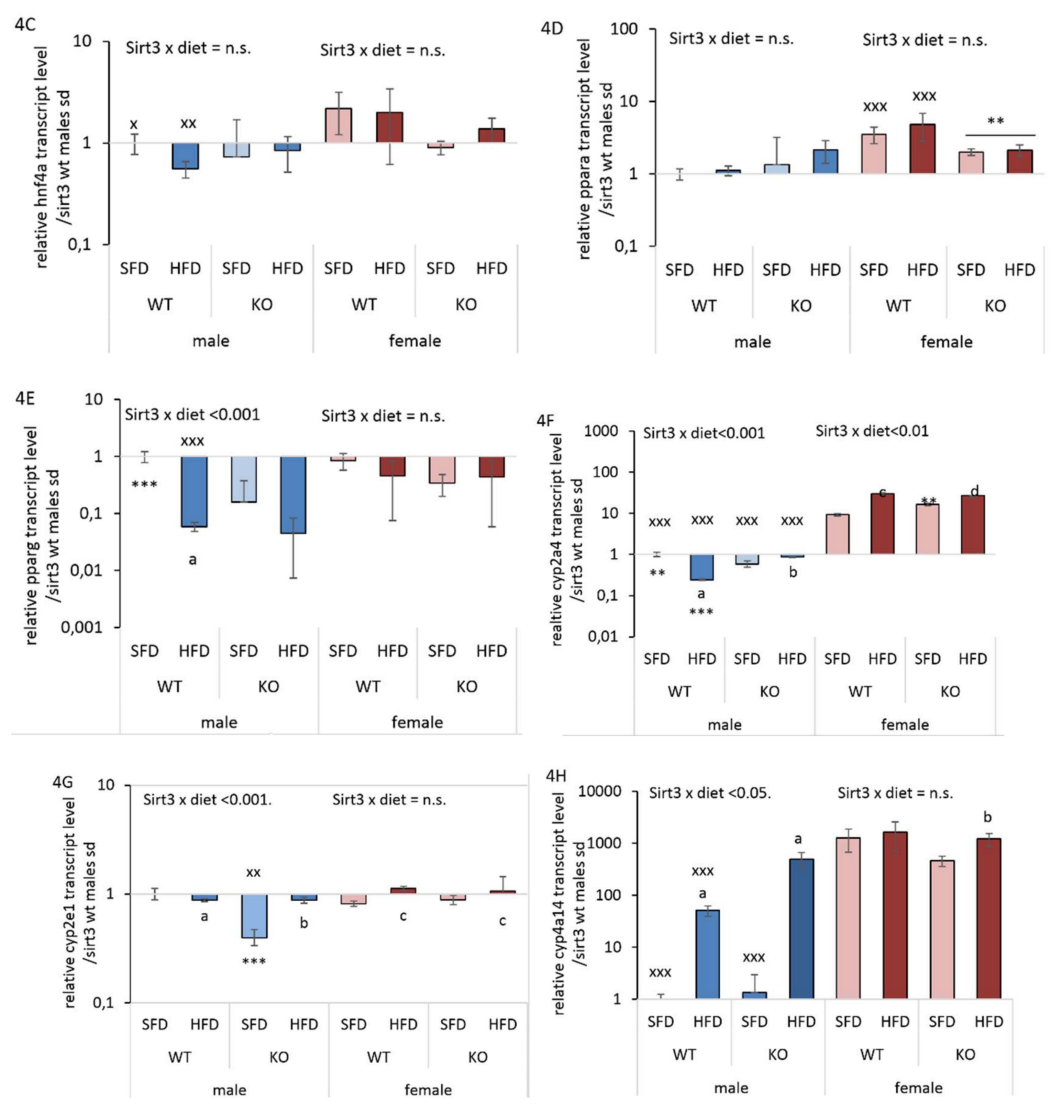
Gene expression of *hnf4α*, *pparα*, *pparγ*, *cyp2a4, cyp2e1*, and *cyp4a14* in the livers of Sirt3 wild-type (WT) and knockout (KO) mice fed with a standard fat diet (SFD) or a high fat diet (HFD) for 10 weeks. (**A**) A heatmap of the mRNA levels of *hnf4α*, *pparα*, *pparγ*, *cyp2a4*, *cyp2e1*, and (**B**) *cyp4a14* genes. The color of the squares on the heat map corresponds to the mean value of the log fold change from three biological and three technical replicates. (**C**) A graphical display of *hnf4a* mRNA level. Males: no changes. Females: no changes. Males vs. females: SFD-fed (^x^ *p* < 0.05) and HFD-fed (^xx^ *p* < 0.01). (**D**) A graphical display of *pparα* mRNA levels. Males: no changes. Females: KO vs. WT mice (** *p* < 0.01). Males vs. females: WT mice (^xxx^ *p* < 0.001). (**E**) A graphical display of *pparγ* mRNA levels. Males: HFD-fed vs. SFD-fed WT mice (^a^ *p* < 0.001); SFD-fed WT vs. KO mice (*** *p* < 0.001). Females: no changes. Males vs. females: HFD-fed WT mice (^xxx^ *p* < 0.001). (**F**) A graphical display of *cyp2a4* mRNA levels. Males: HFD-fed vs. SFD-fed WT mice (^a^ *p* < 0.001); HFD-fed vs. SFD-fed KO mice (^b^ *p* < 0.01); SFD-fed WT vs. KO mice (** *p* < 0.01) and HFD-fed WT vs. KO mice (*** *p* < 0.001); Females: HFD-fed vs. SFD-fed WT (^c^ *p* < 0.001) and KO mice (^d^ *p* < 0.01); SFD-fed KO vs. WT mice (** *p* < 0.01); Males vs. females: ^xxx^ *p* < 0.001. (**G**) A graphical display of *cyp2e1* mRNA levels. Males: HFD-fed vs. SFD-fed WT mice (^a^ *p* < 0.05); HFD-fed vs. SFD-fed KO mice (^b^ *p* < 0.001); SFD-fed KO vs. WT mice (*** *p* < 0.001). Females: HFD-fed vs. SFD-fed mice (^c^ *p* < 0.01). Males vs. females: SFD-fed KO mice (^xx^ *p* < 0.01). (**H**) A graphical display of *cyp4a14* mRNA levels. Males: HFD-fed vs. SFD-fed mice (^a^ *p* < 0.001). Females: HFD-fed vs. SFD-fed KO mice (^b^ *p* < 0.01). Males vs. females: WT mice and SFD-fed KO mice (^xxx^ *p* < 0.001). β-actin was used for normalization. The data are shown as mean ± SD. *n* = 3 per group in technical triplicates.

Hepatocyte nuclear factor 4α (hnf4α) is a master regulator of many genes involved in lipid, glucose, and drug metabolism. In our study, no change in *hnf4a* transcript level was found either within the male or female group of mice. The only differences were found between WT males and females, where males had less *hnf4α* transcript compared to females (Figure 4A,C).

The peroxisome proliferator-activated receptor α (pparα) is shown to be upregulated during HFD as it serves as a ligand for free fatty acids [25]. In males, we observed no change in the transcription level of *pparα*, while in females, *pparα* was reduced in the absence of Sirt3. Interestingly, HFD had no effect on the *pparα* in any sex. WT females had higher *pparα* than males. These data indicate sex-related differences in *pparα* gene expression that are lost in the absence of Sirt3 (Figure 4A,D).

Hepatic *pparγ* gene expression is upregulated in animal models of severe obesity and lipoatrophy [26]. Since in our study HFD-fed mice did not have increased weight gain compared to SFD, we checked whether this was associated with the altered expression of *pparγ* in liver. In WT males, an HFD strongly reduced *pparγ*, which was maintained in KO males as well. On the contrary, females exhibited no change in *pparγ*. Overall, HFD-fed WT males had reduced *pparγ* compared to females. These data indicate that both HFD and Sirt3 depletion reduce *pparγ* only in males (Figure 4A,E).

Cyp2a4 (steroid 15α-hydroxylase) is the enzyme that is constitutively expressed in the livers of female mice while in males its expression is hormonally regulated [27]. Considering its sex-related expression and regulation, we wanted to check whether lack of Sirt3 or change in diet affects the expression of this gene. In WT males, an HFD reduced *cyp2a4*, whereas in KO mice, an HFD rescued the reduced *cyp2a4* observed in SFD-fed mice. In SFD conditions, WT males had more *cyp2a4*, and in HFD conditions less than KO males. In females, HFD induced *cyp2a4*. Additionally, higher *cyp2a4* was observed in SFD-fed KO females compared to WT. Overall, males had lower *cyp2a4* than females across all groups (Figure 4A,F).

Cyp2e1 expression and activity in the liver are increased in humans and in animal models of NAFLD [7,10,11]. Similar to *cyp2a4*, in WT males, an HFD reduced *cyp2e1*, whereas in KO mice an HFD rescued reduced *cyp2e1* observed in SFD-fed mice. SFD-fed KO males displayed lower *cyp2e1* than WT mice. In females, HFD induced *cyp2e1*. The differences in *cyp2e1* between males and females were observed only in SFD-fed KO mice, with lower *cyp2e1* expression in males (Figure 4A,G).

Mouse Cyp4a14 catalyzes the omega-hydroxylation of saturated and unsaturated fatty acids in mice and shows female-predominant expression in liver, where it is induced by pparα [28]. In WT males, HFD strongly increased otherwise very low *cyp4a14* by 51-fold, and in KO males by almost 400-fold. In females, HFD upregulated *cyp4a14* by 2.5-fold in KO mice. Overall, females displayed higher *cyp4a14* expression than males, except in HFD-fed KO mice (Figure 4B,H).

### 3.6. Sirt3 KO Mice Exhibit Increased Protein Oxidative Damage and Upregulated Keap1-Nrf2-Ho1 Axis in Liver

**Figure 5:**
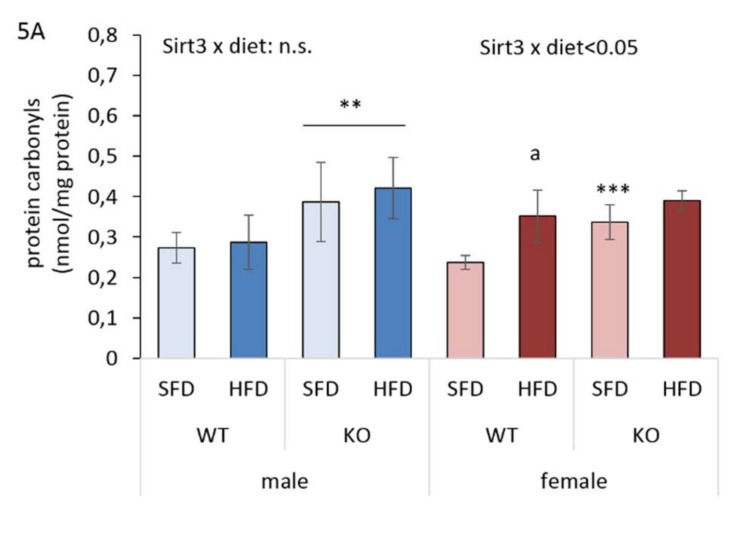

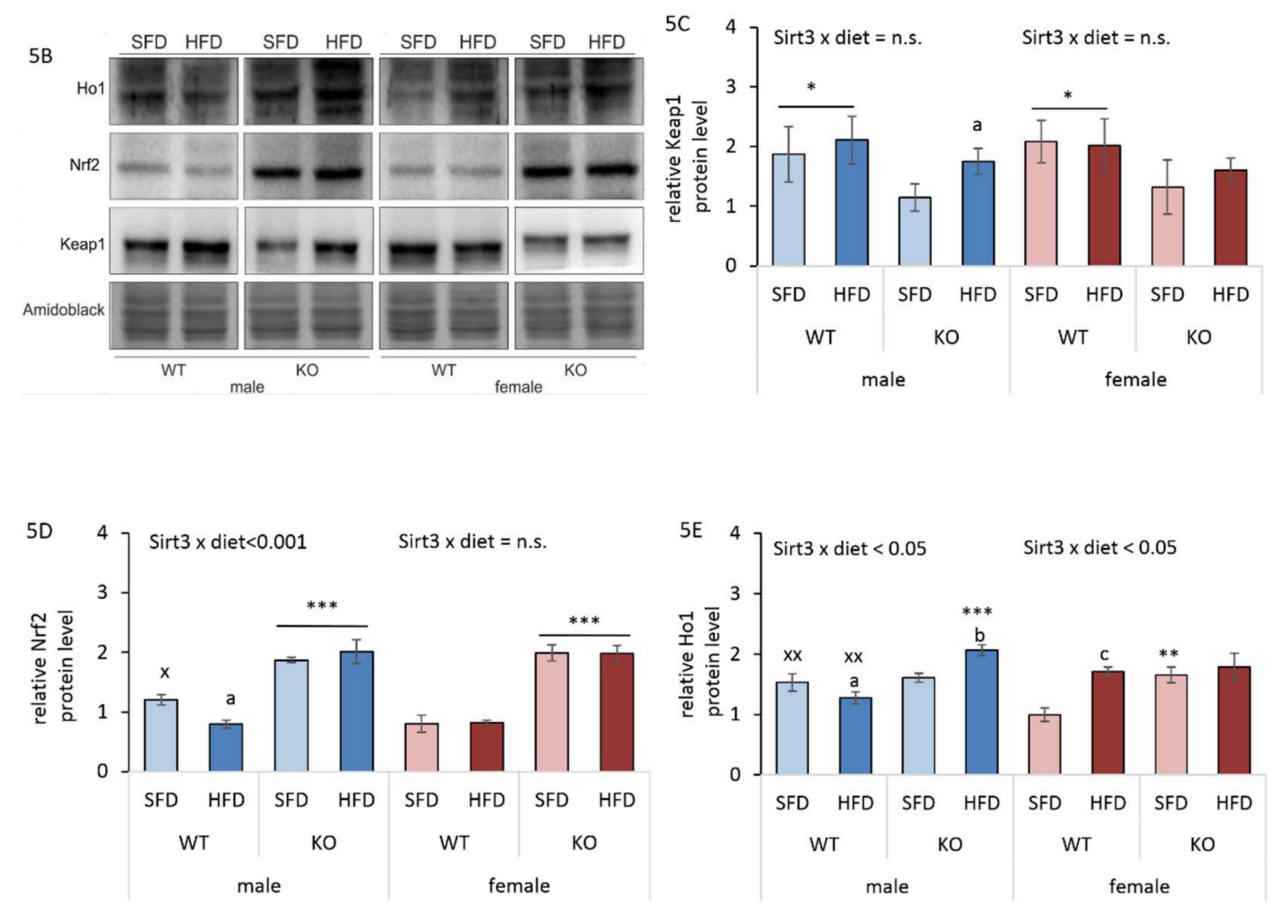
Protein oxidative damage and antioxidant response in the livers of Sirt3 wild-type (WT) and knockout (KO) mice fed with a standard fat diet (SFD) or a high fat diet (HFD) for 10 weeks. (**A**) The total amount of protein carbonyls (PC) measured with an ELISA-based assay at 450 nm. Males: KO vs. WT mice (** *p* < 0.01). Females: HFD-fed vs. SFD-fed WT mice (^a^ *p* < 0.01); SFD-fed KO vs. WT mice (*** *p* < 0.001). Males vs. females: no changes. The data are shown as mean ± SD. *n* = 6 per group. (**B**) The representative immunoblots of hepatic Keap1, Nrf2, and Ho1 protein expression. Amidoblack was used as a loading control. (**C**) A graphical display of the averaged densitometry values for Keap-1 protein expression. Males: HFD-fed vs. SFD-fed KO mice (^a^ *p* < 0.05); WT vs. KO mice (* *p* < 0.05). Females: WT vs. KO mice (* *p* < 0.05). Males vs. females: no changes. (**D**) A graphical display of the averaged densitometry values for Nrf-2 protein expression. Males: HFD-fed vs. SFD-fed WT mice (^a^ *p* < 0.001); KO vs. WT mice (*** *p* < 0.001). Females: KO vs. WT mice (*** *p* < 0.001). Males vs. females: SFD-fed WT mice (^x^ *p* < 0.05). (**E**) A graphical display of averaged densitometry values of Ho-1 protein expression. Males: HFD-fed vs. SFD-fed WT mice (^a^ *p* < 0.05); HFD-fed vs. SFD-fed KO mice (^b^ *p* < 0.01); HFD-fed KO vs. WT mice (*** *p* < 0.001). Females: HFD-fed vs. SFD-fed WT mice (^c^ *p* = 0.002); SFD-fed KO vs. WT mice (** *p* < 0.01). Males vs. females: WT mice (^xx^ *p* < 0.05). The data are shown as mean ± SD. *n* = 6.

Following the observed differences between males and females in lipid accumulation and the genes involved in lipid homeostasis, along with the fact that sensitivity towards the oxidative stress is sex-related as well [29,30], we next examined sensitivity to oxidative stress with respect to Sirt3 or diet by measuring protein carbonylation (PC), a marker of protein oxidative damage. In males, higher PC levels were observed in the absence of Sirt3. Contrary to that, in WT females, an HFD increased PC, resulting in levels similar to KO females in both diets. Moreover, SFD-fed KO females had higher PC than WT mice. Overall, the sex-specific differences evident in HFD-fed WT mice were abrogated in the KO mice, where similar PC levels were observed in both sexes (Figure 5A). These data indicate that, besides protein oxidative damage caused by the absence of Sirt3 in both sexes, females are also susceptible to protein damage caused by an HFD only.

We next aimed to investigate whether major proteins involved in antioxidative response pathway, such as Keap1/Nrf2/Ho1, were altered. Nrf2 is a transcription factor that induces the gene expression of antioxidant enzymes and many other cytoprotective enzymes. Upon oxidative stress, the interaction between Keap1 and Nrf2 is disrupted, which induces Nrf2-dependent gene expression [31]. WT males had higher Keap1 compared to KO mice and HFD partially rescued Keap1 in KO mice. Likewise, in females, Keap1 was also reduced in KO mice. There were no differences in Keap1 between males and females (Figure 5C). In WT males, HFD reduced Nrf2. Moreover, Nrf2 was higher in KO mice compared to WT mice of both sexes, which is in accordance with reduced Keap1. Differences between males and females were evident only in SFD-fed WT mice, where males had higher Nrf2 (Figure 5D). In males, an HFD had the opposite effect on Ho1 expression, where it decreased the expression of the Ho1 protein in WT and increased it in KO males. In WT females, an HFD increased Ho-1 protein level, which remained higher in KO mice, irrespective of diet. Differences in Ho1 between males and females were only observable in WT mice, with higher Ho1 in SFD-fed and lower in HFD-fed males (Figure 5E). This finding indicates the presence of the adaptive stress response in conditions of nutritional excess only in the absence of Sirt3.

### 3.7. HFD-Induced Reduction in Sirt3/MnSOD Axis is Male-Specific

**Figure 6:**
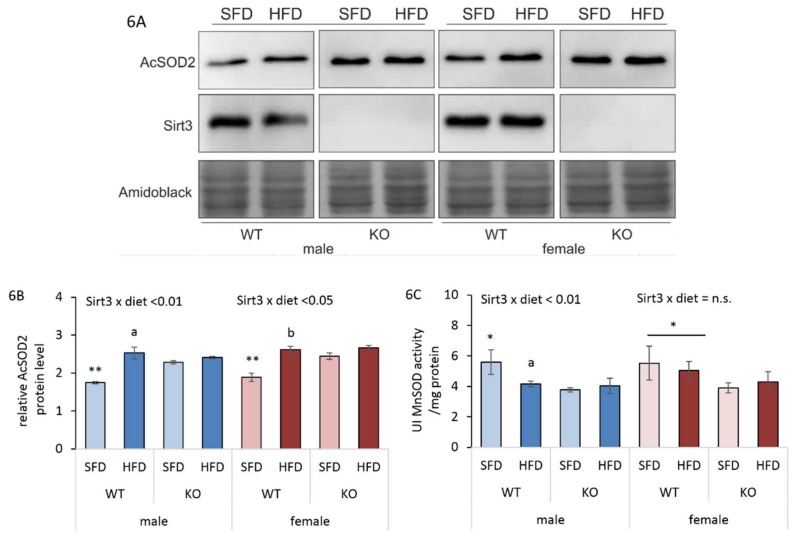
Sirt3/MnSOD axis in liver of Sirt3 wild-type (WT) and knockout (KO) mice fed with a standard fat diet (SFD) or a high fat diet (HFD) for 10 weeks. (**A**) A representative immunoblot of hepatic Sirt3 and AcSOD2 protein expression. Amidoblack was used as a loading control. (**B**) A graphical display of averaged densitometry values for AcSOD2 protein expression. Males: HFD-fed vs. SFD-fed WT mice (^a^ *p* < 0.01); SFD-fed WT vs. KO mice (** *p* < 0.01). Females: HFD-fed vs. SFD-fed WT mice (^b^ *p* < 0.01); SFD-fed WT vs. KO mice (** *p* < 0.01). Males vs. females: no change. (**C**) MnSOD activity. Males: HFD-fed vs. SFD-fed WT mice (^a^ *p* < 0.01); SFD-fed WT vs. KO mice (* *p* < 0.05). Females: WT vs. KO mice (* *p* < 0.05). Males vs. females: no change. The data are shown as mean ± SD. *n* = 6 per group.

Sirt3 mediates the reduction of oxidative and metabolic damage, while exposure to HFD leads to reduced Sirt3 expression, consequent hyper acetylation of manganese superoxide dismutase (AcSOD2), and the reduction of SOD2 activity (hereafter MnSOD) [32]. Since we found that an HFD reduced Sirt3 protein level only in males, we further examined whether this pattern affects AcSOD2 protein and MnSOD activity in the same way. HFD-fed WT males displayed increased AcSOD2 protein levels along with decreased MnSOD activity. SFD-fed WT males had reduced AcSOD2 protein and higher MnSOD activity than KO mice. HFD-fed WT females also displayed increased AcSOD2 protein, but without change in MnSOD activity. SFD-fed WT females had reduced AcSOD2 protein but higher MnSOD activity on both diets compared to KO mice (Figure 6A–D). This suggests that the acetylation status of MnSOD acetyl-K68 that regulates MnSOD activity in males under HFD conditions, does not contribute to alteration of MnSOD activity in females. Both KO males and females had similar AcSOD2 levels, with reduced MnSOD activity, thus confirming the importance of Sirt3 in regulation of MnSOD activity.

### 3.8. HFD Affects Antioxidant Enzyme Activities Differently in Males and Females

**Figure 7:**
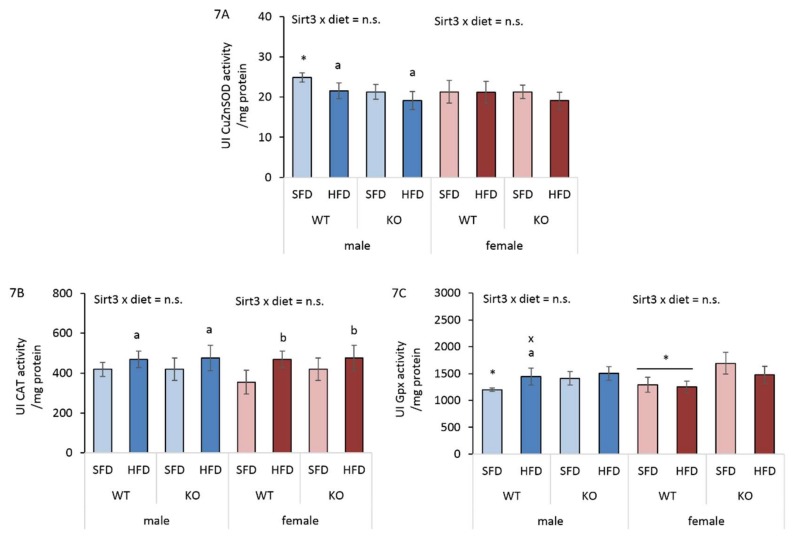
Analysis of CuZnSOD, Cat, and Gpx1 activities in the livers of Sirt3 wild-type (WT) and knockout (KO) mice of both sexes fed with a standard fat diet (SFD) or a high fat diet (HFD) for 10 weeks. (**A**) CuZnSOD activity. Males: HFD-fed vs. SFD-fed mice (^a^ *p* < 0.05); SFD-fed WT vs. KO mice (* *p* < 0.05); Females: no changes. Males vs. females: no changes. (**B**) Cat activity. Males: HFD-fed vs. SFD-fed mice (^a^ *p* < 0.05). Females: HFD-fed vs. SFD-fed mice (^b^ *p* < 0.01). Males vs. females: no changes. (**C**) Gpx1 activity. Males: HFD-fed vs. SFD-fed WT mice (^a^ *p* < 0.05); SFD-fed WT vs. KO mice (* *p* < 0.05). Females: WT vs. KO mice (* *p* < 0.05). Males vs. females: HFD-fed WT mice (^x^ *p* < 0.05). The data are shown as mean ± SD. *n* = 6 per group.

Beside major mitochondrial antioxidant enzyme MnSOD, we also determined other antioxidant enzymes, such as copper zinc superoxide dismutase (CuZnSOD, SOD1), catalase (Cat), and glutathione peroxidase (Gpx1) at protein level (Supplementary Figure S3) and their activities (Figure 7A–C). We observed a discrepancy between protein expression and antioxidant enzyme activity, indicating the complex regulatory mechanisms of enzyme activities. Therefore, the results of enzyme activities are more informative and are shown here. In males, an HFD reduced very mildly CuZnSOD activity in both WT (14%) and KO (10%) mice. Additionally, SFD-fed WT mice had marginally higher CuZnSOD activity compared to KO mice (14.2%). On the contrary, females displayed no changes in CuZnSOD activity (Figure 7A). Cat activity was increased following an HFD in both sexes and genotypes (Figure 7B). The inducing effect of HFD on Cat activity in both genotypes and sexes suggests that increased Cat activity is needed to effectively degrade excess of H_2_O_2_ produced by increased lipid metabolism, irrespective of sex or Sirt3. Gpx is a cytosolic enzyme that also functions in the detoxification of H_2_O_2_, specifically by catalyzing the reduction of hydrogen peroxide to water. In WT males, HFD increased Gpx activity to levels of KO mice. SFD-fed WT males also displayed lower Gpx activity compared to KO mice. In females, Gpx activity was lower in WT mice compared to KO mice. The differences in Gpx activity between males and females were evident only in HFD-fed WT mice, where males had higher Gpx activity (Figure 7C). These results point toward differential sex-related effect of HFD on antioxidant enzyme activities.

### 3.9. HFD and Sirt3 Depletion Affect Mitochondrial Respiration Differently in Males and Females

Sirt3 is an important regulator of mitochondrial respiration, and its targets include subunits of the respiratory chain complexes. Studies showed that KO mice display decreased oxygen consumption in liver [33] and reduced glucose tolerance when placed on an HFD [10]. To examine whether the loss of Sirt3 would impair respiration in a sex- and diet-dependent manner, we measured oxygen consumption in mitochondria from liver using a Clark type electrode.

**Figure 8:**
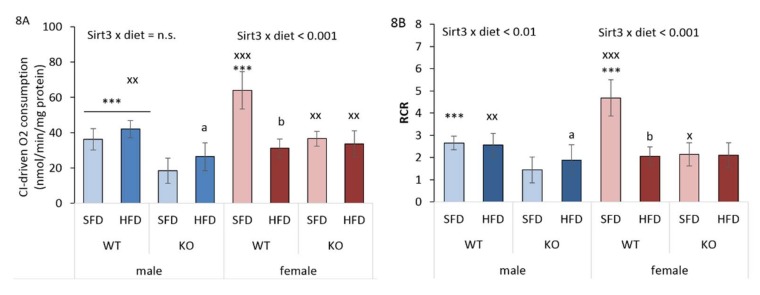
Complex I (CI)-driven respiration and the respiratory control ratio (RCR) of liver mitochondria of Sirt3 wild-type (WT) and knockout (KO) mice of both sexes fed with a standard fat diet (SFD) or a high fat diet (HFD) for 10 weeks. (**A**) CI-driven respiration. Males: HFD-fed vs. SFD-fed KO mice (^a^ *p* < 0.05); WT vs. KO mice (*** *p* < 0.001). Females: HFD-fed vs. SFD-fed WT mice (^b^ *p* < 0.001); SFD-fed WT vs. KO mice (*** *p* < 0.001). Males vs. females: SFD-fed WT mice (^xxx^ *p* < 0.001); HFD-fed WT mice (^xx^ *p* < 0.01); KO mice (^xx^ *p* < 0.01). (**B**) The respiratory control ratio (RCR). Males: HFD-fed vs. SFD-fed KO mice (^a^ *p* < 0.001); SFD-fed WT vs. KO mice (*** *p* < 0.001). Females: HFD-fed vs. SFD-fed WT mice (^b^ *p* < 0.001); SFD-fed WT vs. KO mice (*** *p* < 0.001). Males vs. females: SFD-fed WT mice (^xxx^ *p* < 0.001); HFD-fed WT mice (^xx^p < 0.01); SFD-fed KO mice (^x^ *p* < 0.05). The data are shown as mean ± SD. *n* = 4 per group.

WT males had higher malate/glutamate (MG) + ADP respiration than KO males, suggesting defective CI-driven respiration in the absence of Sirt3. Interestingly, an HFD partially restored low CI-driven respiration in KO males. In WT females, an HFD significantly decreased CI-driven respiration, which remained low in the absence of Sirt3. Finally, we observed that females had higher CI-driven respiration compared to males, except in HFD-fed WT mice, where this difference was reversed in favor of males (Figure 8A).

The respiratory control ratio (RCR) is defined as the ratio of the state 4 respiratory rate (termination of ATP synthesis by addition of oligomycin) to the state 3 (ADP-stimulated respiration) respiratory rate and indicates how well the electron transport system is coupled to ATP synthesis. WT males had higher RCR than KO males on a SFD, while an HFD partially restored low the RCR observed in the absence of Sirt3. In WT females, an HFD decreased RCR, which remained low in the absence of Sirt3. Females had higher RCR than males in SFD-fed conditions, while the opposite was found in HFD-fed WT mice. These results collectively indicate that the effective CI respiration in both sexes depends on Sirt3. Moreover, both CI-driven respiration and RCR display a sex-specific effect of HFD and Sirt3, with an inducing effect of HFD in KO males and a suppressive effect in WT females.

## 4. Discussion

Sexual dimorphism exists in various physiological processes, with males and females differing with respect to their regulation of energy homeostasis, metabolic rate, or body weight gain [34]. For example, HFD feeding leads to larger body weight gain in male rats/mice than in females [35]. In this study, we report the following novel observations in conditions of HFD feeding and Sirt3 depletion in liver of 129S mice: (a) HFD reduces hepatic Sirt3 expression solely in males, but only partially; (b) HFD-induced lipid accumulation is alleviated in the absence of Sirt3 only in males, which may be attributed to impaired glucose uptake and an increased reliance on fatty acids; (c) in females, either an HFD or the absence of Sirt3 compromised mitochondrial respiration and increased protein oxidative damage, which could be associated with more efficient lipid metabolism.

Sirt3 plays an important role in regulating lipid homeostasis by ameliorating HFD-induced inflammation, liver fibrosis, and steatosis. Reports show that HFD feeding in WT male mice results in a reduction of Sirt3 expression in the liver [36] and that deleterious effects of HFD feeding are exacerbated in male mice lacking Sirt3 [10]. Accordingly, we found that Sirt3 was reduced upon HFD feeding in male mice, however, without any change in females (Figure 1A,B). This result suggests that the regulation of Sirt3 in a sexually dimorphic manner following HFD in males may contribute to the observed sex-related differences in the development of metabolic dysregulation.

It has been shown that a decreased level of Sirt3 contributes to impaired glucose metabolism [24]. However, in our study, we did not observe the effect of Sirt3 on differences in glucose levels, only the effect of sex, where females had lower fasting glucose levels compared to males (Figure 2B), suggesting a possible role of female sex hormones in maintaining low glucose in females [37]. The interesting fact is that we did not observe gained weight in mice fed with an HFD (Figure 2A). The observed phenotype upon HFD feeding comes from the specific characteristics of this particular strain of mice (129S), which are not prone to the development of obesity when fed with an HFD [38]. However, despite the fact that mice did not gain weight following HFD feeding, both sexes displayed excessive hepatic lipid accumulation (Figure 3), indicating a fatty liver phenotype after ten weeks of an HFD.

Sirt3 deficiency reduces fatty acid oxidation and results in the accumulation of hepatic lipids in SFD-fed male mice [39], which is confirmed in our study. On the contrary, this effect was not evident in Sirt3-depleted females, which showed less accumulation of lipids than males in SFD conditions (Figure 3I). Thus, disturbances in fatty acid oxidation in standard conditions and upon the depletion of Sirt3 may result in elevated lipids and impaired metabolism, thus supporting the critical role of Sirt3 in the maintenance of metabolic homeostasis in males. Studies have shown that an HFD acts as a metabolic stressor causing mitochondrial dysfunction and other metabolic changes that contribute to pathological conditions [4]. In HFD conditions, Sirt3-depleted males displayed the lowest total lipid content in liver along with the lowest hepatic glucose uptake (Figure 3I,J). These results indicate that in the absence of Sirt3 in males, HFD may cause impairments in hepatic glucose uptake, creating an increased reliance on fatty acids. This was previously also shown in the skeletal muscles of Sirt3 KO male mice [33]. Again, the observed differences in males were not evident in Sirt3-depleted females. The absence of these effects in WT mice indicates that sex-related differences in lipid accumulation and glucose uptake are present only in Sirt3-depleted mice.

In tissues that have a high fatty acid uptake, such as adipocytes and liver tissue, the capacity for the β oxidation of fatty acids, the major lipid catabolic pathways, is regulated at the level of gene expression in response to various physiologic stimuli [28]. Moreover, the maintenance of lipid homeostasis relies on cytochrome P450 (P450) enzymes, the majority of which are regulated in a sex-dependent manner [40]. To test if hepatic P450 enzymes are involved in sex-related differences in lipid accumulation in the absence of Sirt3, we measured the expression of several genes involved in fatty acid oxidation and lipid homeostasis (Figure 4). One important factor is *pparγ*, which is predominantly expressed in adipose tissue but also in the liver and muscle, and promotes the storage of lipids [41]. Since in our study HFD-fed mice did not have increased weight compared to SFD-fed mice, we tested whether this was due to the altered expression of *pparγ*, which is essential for adipogenesis [42]. Indeed, both sexes displayed downregulated levels of *pparγ* (Figure 4E), allowing us to hypothesize that the downregulation of hepatic *pparγ* may be related to the fact that mice did not gain weight after feeding with HFD. Furthermore, we observed sex-related differences in *cyp2e1, cyp2a4,* and *cyp4a14* transcripts of P450 genes involved in the maintenance of lipid homeostasis (Figure 4A,F–H). Females generally responded to an HFD with an upregulated expression of these involved genes, while males had a different response to an HFD with respect to Sirt3. Considering their role in lipid metabolism, the downregulation of *cyp2a4* and *cyp2e1* in male KO mice fed with SFD may be associated with their tendency towards accumulating more hepatic fat than females. In HFD conditions, these genes tend to be upregulated in KO males, therefore suggesting more effective lipid metabolism in association with lower accumulation of total lipids in liver.

Since we found sex-related differences in several parameters involved in lipid homeostasis upon different types of diet, we next investigated whether they reflected sex-related sensitivity to oxidative stress under the same conditions. Protein carbonylation (PC) is considered to be an important marker of oxidative stress resulting from HFD-induced oxidative damage to proteins [43,44]. Here, we show that an HFD induced a female-specific increase in the PC of WT mice (Figure 5A) as a possible consequence of the upregulated expression of genes involved in fatty acid oxidation, that is associated with the generation of hydrogen peroxide [45] involved in protein oxidative damage. Unlike WT males, HFD-fed WT females also displayed increased Ho1 protein expression (Figure 5B,E), which is an indicator of the presence of oxidative stress [46]. However, they failed to counteract oxidative damage by the activation of an antioxidative response, since no induction of the Keap1/Nrf2 axis (Figure 5C,D) and major antioxidative enzyme activities (MnSOD, CuZnSOD, Gpx1) was observed (Figure 6C and Figure 7A,C), resulting in greater protein oxidative damage (Figure 5A). On the other hand, higher PC levels in KO mice of both sexes were associated with upregulated Keap1/Nrf2 axis, but with no response in antioxidant enzyme activities. This shows that in WT mice, an HFD induces oxidative stress only in females, pointing at their increased susceptibility towards the oxidative stress in conditions of nutritive stress. Furthermore, Sirt3 deficiency increases susceptibility to protein oxidative damage equally in both sexes, indicating that Sirt3 is important for protection against the oxidative damage of proteins.

Several studies demonstrated that lipid metabolism has a potential to generate ROS [47,48]. For example, both peroxisomal and mitochondrial β-oxidation produce H_2_O_2_ and O_2_^-^ as byproducts of fatty acid degradation. During nutritional excess, the imbalance in lipid metabolism along with upregulated key genes involved in fatty acid β-oxidation, such as *pparα*, contributes to increased ROS and oxidative stress [45]. Catalase (Cat), a peroxisomal enzyme, has also been found in cardiac mitochondria with significantly increased activity during HFD feeding [48]. We observed the inducing effect of HFD on Cat activity in both genotypes and sexes (Figure 7B), suggesting that increased Cat activity is needed to effectively degrade excess of H_2_O_2_ produced by increased fat metabolism in HFD-fed mice.

Mitochondrial function is tightly associated with the activity of the respiratory chain complexes, and depends on the degree of coupling of oxidative phosphorylation [49]. Fatty acids are metabolic fuels and β-oxidation represents their main degradation pathway in mitochondria [50]. Sirt3 WT females had significantly higher CI-driven respiration and RCR than Sirt3 WT males in standard conditions, suggesting a more efficient function of Complex I in the electron transport chain of hepatic mitochondria in females. Contrary to males, in WT females, HFD feeding dramatically decreased MG-ADP state respiration, indicating female-specific impairment in CI-driven respiration and RCR following an HFD (Figure 8A,B). The observed defective CI-driven respiration in HFD-fed females could be due to their higher fatty acid oxidation, in synchronization with upregulated genes involved in the lipid oxidation process (*pparα*, *cyp2a4*, *cyp2e1*, *cyp4a14*), resulting in increased oxidative damage of mitochondrial proteins, thereby affecting mitochondrial respiration. Indeed, fatty acids can act as inhibitors of mitochondrial respiration, either by the partial inhibition of electron transport within CI or III, or by a decrease in proton motive force [51,52]. Based on this result, we hypothesize that in conditions of nutritive excess, more efficient lipid metabolism in WT females may cause higher oxidative stress in association with reduced mitochondrial function.

Sirt3 KO male mice display decreased CI-driven oxygen consumption [33], which is in agreement with our results. In addition, we show for the first time that, despite reduced respiration in Sirt3-depleted mice, females have higher respiration than males. However, since in KO males an HFD increased respiration, we hypothesize that they compensate impaired glucose uptake by increasing their reliance on fatty acids to provide substrates for the respiratory chain. This is in accordance to [53,54], where HFD-fed male rodents exhibited increased mitochondrial capacity and respiration [55]. Our results collectively indicate that the CI-driven respiration displays a sex-specific effect with respect to both HFD and Sirt3, with an inducing effect of HFD on respiration in KO males and a suppressive effect in WT females.

Sexual dimorphism exists in various physiological processes, which also includes sex-related prevalence towards metabolic dysregulation. In this study, we found significant sex differences at the level of metabolic, oxidant/antioxidant, and mitochondrial parameters. In addition, this study points towards a different role of Sirt3 in males and females under the conditions of nutritive stress. This is an important step that adds to previous knowledge that can be studied to prevent metabolic dysfunction, improve preclinical research, and allow for the development of sex-related therapeutic agents for obesity and diabetes.

## Supplementary Materials

The following are available online at https://www.mdpi.com/2076-3921/9/2/174/s1, Figure S1: Body weight gain of Sirt3 WT and KO mice fed with standard fat diet (SFD) or high fat diet (HFD) for 10 weeks. Figure S2: Quantification of hepatic lipid accumulation signal (from Figure S2A–H) in Sirt3 WT and KO mice of both sexes fed with standard fat diet (SFD) or high fat diet (HFD) for 10 weeks. Figure S3: Western blot analysis of antioxidative enzymes in Sirt3 WT and KO mice of both sexes fed with standard fat diet (SFD) or high fat diet (HFD) for 10 weeks. Table S1: Assays (Taqman^®^ Applied Biosystems, Foster City, CA, USA) used for real time quantitative PCR analyses, Table S2: Antibodies used in this study for the Western blot analyses.
