# Supplementary material for: Role of Sirt3 in Differential Sex-Related Responses to a High-Fat Diet in Mice
Source: Antioxidants (Basel). 2020 Feb 20;9(2):174. doi: 10.3390/antiox9020174 (PMC7071037; doi:10.3390/antiox9020174)
Supplement: Supplementary file 1 [file antioxidants-09-00174-s001.pdf]

antioxidants

MDPI

Table S1. Assays (Taqman® Applied Biosystems, Foster City, CA, USA) used for real time quantitative PCR analyses.

Gene	Assay ID	Product Size (bp)
β-actin	Mm00607939_s1	115
cyp2a4	Mm00487248_m1	75
cyp2e1	Mm00491127_m1	83
cyp4a14	Mm00484132_m1	71
hnf4α	Mm00433964_m1	114
pparα	Mm00627559_m1	86
pparγ	Mm00440940_m1	63

Table S2. Antibodies used in this study for the Western blot analyses.

Antibody	Dilution	Host	Manufacturer
Sirt3 (D22A3)	1:1000	Rabbit	Cell Signaling Technology, Danvers, MA, USA
Ho-1 (ab52947)	1:700	Rabbit	Abcam, Cambridge, UK
Nrf2 (ab31163)	1:2000	Rabbit	Abcam, Cambridge, UK
Keap1 (60027-1-Ig)	1:1000	Mouse	Proteintech, Rosemont, IL, USA
AcSOD2 (ab137037)	1:1000	Rabbit	Abcam, Cambridge, UK
CuZnSOD (ab 16831)	1:3000	Rabbit	Abcam, Cambridge, UK
Cat (ab1877)	1:4000	Rabbit	Abcam, Cambridge, UK
Gpx1 (ab16798)	1:2000	Rabbit	Abcam, Cambridge, UK
Anti-mouse (170-6516)	1:5000	Goat	Bio-rad, Hercules, CA, USA
Anti-rabbit (NA934)	1:5000	Goat	GE Healthcare, Chicago, IL, USA

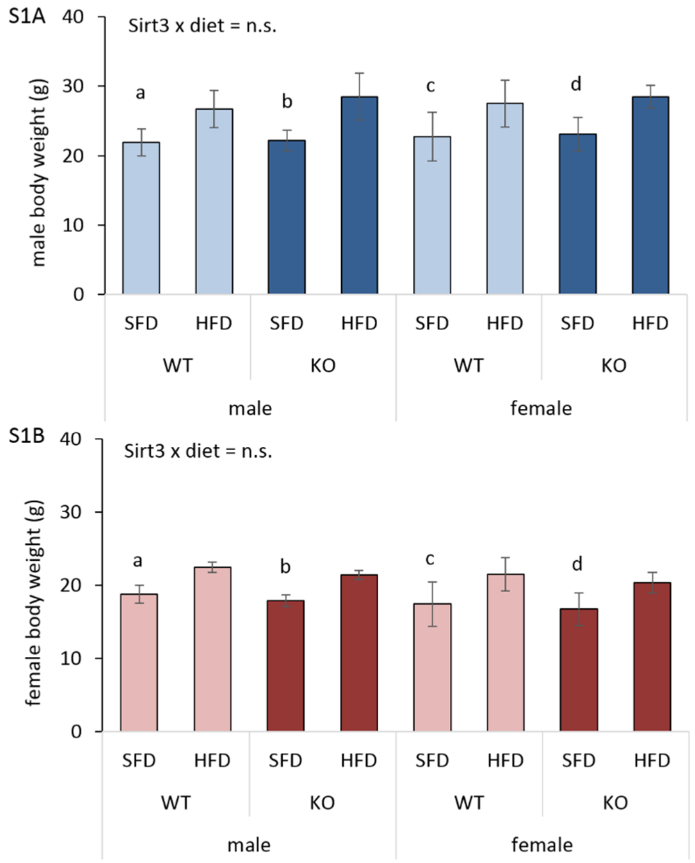

Figure S1. Body weight gain of Sirt3 WT and KO mice fed with standard fat diet (SFD) or high fat diet (HFD) for 10 weeks. **(A)** Body weight of male mice. Week 1 vs. week 10 in SFD-fed WT ($^{a}p<0.01$), HFD-fed WT ($^{b}p<0.001$), SFD-fed KO ($^{c}p<0.05$) and HFD-fed KO mice ($^{d}p<0.001$). **(B)** Body weight of female mice. Week 1 vs. week 10 in SFD-fed WT ($^{a}p<0.001$), HFD-fed WT ($^{b}p<0.001$), SFD-fed KO ($^{c}p<0.05$) and HFD-fed KO mice ($^{d}p<0.01$). Data are shown as mean ± SD. N=6 per group.

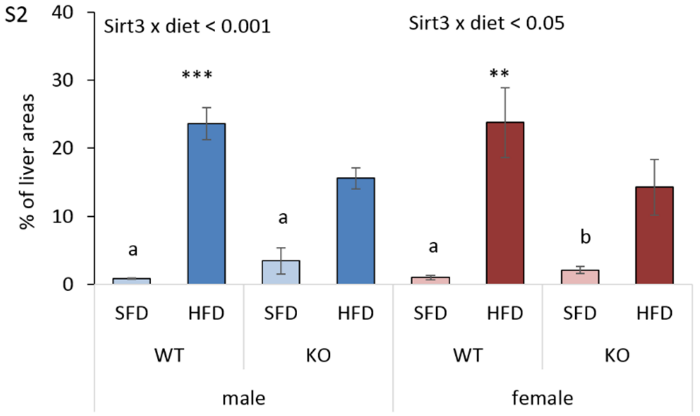

Figure S2. Quantification of hepatic lipid accumulation signal (from Figure 2A-H) in Sirt3 WT and KO mice of both sexes fed with standard fat diet (SFD) or high fat diet (HFD) for 10 weeks. **Males**: WT and KO mice (ap<0.001); HFD-fed WT vs. KO mice (***p<0.001). **Females**: SFD-fed vs. HFD-fed WT mice (ap<0.001); SFD-fed vs. HFD-fed KO mice (bp<0.01); HFD-fed WT vs. KO mice (**p<0.01). **Males vs. females**: no changes. Data are shown as mean ± SD. N=3 per group.

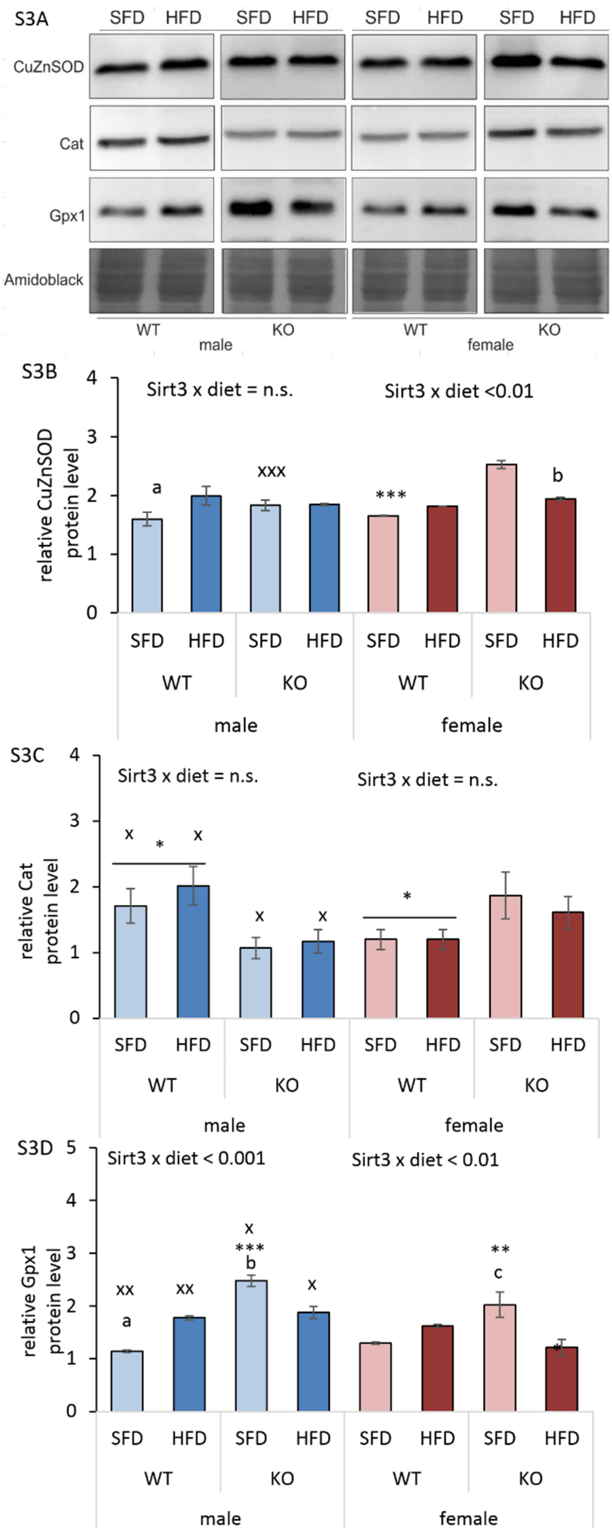

Figure S3. Western blot analysis of antioxidative enzymes in Sirt3 WT and KO mice of both sexes fed with standard fat diet (SFD) or high fat diet (HFD) for 10 weeks. **(A)** Representative immunoblot of hepatic CuZnSOD, Cat and Gpx1 protein expression. Amidoblack was used as a loading control. **(B)** Graphical display of averaged densitometry values of CuZnSOD protein expression. **Males**: SFD-fed vs. HFD-fed WT mice ([a]$p<0.05$). **Females**: HFD-fed vs. SFD-fed KO mice ([b]$p<0.001$); SFD-fed WT vs. KO mice (***$p<0.001$). **Males vs. females**: SFD-fed KO mice ([xxx]$p<0.001$). **(C)** Graphical display of averaged densitometry values of Cat protein expression. **Males**: WT vs. KO mice (*$p<0.05$). **Females**: WT vs. KO mice (*$p<0.05$). **Males vs. females**: WT mice ([x]$p<0.05$) and KO mice ([+]$p<0.05$). **(D)** Graphical display of averaged densitometry values of Gpx1 protein expression. **Males**: SFD-fed vs. HFD-fed

WT mice ([a]p<0.001); SFD-fed vs. HFD-fed KO mice ([b]p<0.01); SFD-fed KO vs. WT mice (***p<0.001). **Females**: SFD-fed vs. HFD-fed KO mice ([c]p<0.01); SFD-fed KO vs. WT mice (**p<0.01); HFD-fed KO vs. WT mice (*p<0.05). **Males vs. females**: SFD-fed WT mice ([xx]p<0.01); HFD-fed WT ([xx]p<0.01), SFD-fed KO ([x]p<0.05) and HFD-fed KO mice ([x]p<0.05). Data for B), C) and D) are shown as mean ± SD. N=4 per group.
